# Acute Skin Damage and Late Radiation-Induced Fibrosis and Inflammation in Murine Ears after High-Dose Irradiation

**DOI:** 10.3390/cancers11050727

**Published:** 2019-05-25

**Authors:** Annique C. Dombrowsky, Jannis Schauer, Matthias Sammer, Andreas Blutke, Dietrich W. M. Walsh, Benjamin Schwarz, Stefan Bartzsch, Annette Feuchtinger, Judith Reindl, Stephanie E. Combs, Günther Dollinger, Thomas E. Schmid

**Affiliations:** 1 Institute for Radiation Medicine, Helmholtz Zentrum München GmbH, 85764 Neuherberg, Germany; annique.hunger@helmholtz-muenchen.de (A.C.D.); stefan.bartzsch@helmholtz-muenchen.de (S.B.); stephanie.combs@helmholtz-muenchen.de (S.E.C.); 2 Department of Radiation Oncology, Technical University of Munich, School of Medicine, Klinikum rechts der Isar, 81675 Munich, Germany; 3 Institute for Applied Physics and Metrology, Universität der Bundeswehr München, 85577 Neubiberg, Germany; jannis.schauer@unibw.de (J.S.); matthias.sammer@unibw.de (M.S.); d.walsh@dkfz-heidelberg.de (D.W.M.W.); benjamin.schwarz@unibw.de (B.S.); judith.reindl@unibw.de (J.R.); guenther.dollinger@unibw.de (G.D.); 4 Research Unit Analytical Pathology, Helmholtz Zentrum München GmbH, 85764 Neuherberg, Germany; andreas.parzefall@helmholtz-muenchen.de (A.B.); annette.feuchtinger@helmholtz-muenchen.de (A.F.); 5 German Cancer Consortium, 69120 Heidelberg, Germany; 6 Clinical Cooperation Unit Translational Radiation Oncology, Heidelberg Ion Therapy Center (HIT), Heidelberg Institute of Radiation Oncology Heidelberg University Medical School and National Center for Tumor Diseases, German Cancer Research Center, 69120 Heidelberg, Germany

**Keywords:** hypofractionation, side effects, acute, late, high-dose, fractionated radiotherapy, skin

## Abstract

The use of different scoring systems for radiation-induced toxicity limits comparability between studies. We examined dose-dependent tissue alterations following hypofractionated X-ray irradiation and evaluated their use as scoring criteria. Four dose fractions (0, 5, 10, 20, 30 Gy/fraction) were applied daily to ear pinnae. Acute effects (ear thickness, erythema, desquamation) were monitored for 92 days after fraction 1. Late effects (chronic inflammation, fibrosis) and the presence of transforming growth factor beta 1 (TGFβ1)-expressing cells were quantified on day 92. The maximum ear thickness displayed a significant positive correlation with fractional dose. Increased ear thickness and erythema occurred simultaneously, followed by desquamation from day 10 onwards. A significant dose-dependency was observed for the severity of erythema, but not for desquamation. After 4 × 20 and 4 × 30 Gy, inflammation was significantly increased on day 92, whereas fibrosis and the abundance of TGFβ1-expressing cells were only marginally increased after 4 × 30 Gy. Ear thickness significantly correlated with the severity of inflammation and fibrosis on day 92, but not with the number of TGFβ1-expressing cells. Fibrosis correlated significantly with inflammation and fractional dose. In conclusion, the parameter of ear thickness can be used as an objective, numerical and dose-dependent quantification criterion to characterize the severity of acute toxicity and allow for the prediction of late effects.

## 1. Introduction

At least half of all cancer patients receive ionizing radiation as part of their treatment [1,2,3]. Despite the effective tumor control of radiation therapy, the risk of acute and late side effects remains a main problem [4]. In humans, acute side effects develop within a few weeks of treatment [1] and usually last four to six weeks [4]. Late side effects manifest after a latent period of three months [5] to years [1] and may, in the worst cases, become chronic [4].

Early side effects of radiation therapy typically manifest in tissues with a high proliferative activity, such as the skin or the mucosae of the gastro-intestinal tract [6]. Cells of the basal layer of the epidermis of the skin are rapidly dividing and are therefore very prone to cell death upon radiation therapy [6,7]. Secondary to the death of basal cells, an inflammatory reaction evolves, resulting in an erythematous reaction [8]. Inflammation is the first acute response of the skin [6]. Dry desquamation is caused by fast cell production in the basal layers [9]. Moist desquamation appears after high doses of radiation because cell production in the basal layers cannot compensate for the loss of cells in the basal layers and exudate is released [9,10]. Moreover, hair follicle loss can occur as another early side effect [11]. Tissue regeneration occurs from surviving basal stem cells [10]. In contrast, radiation-induced late side effects are usually irreversible and may be progressive in severity [1]. Late damage of irradiated skin is mainly characterized by atrophy [12] and abnormal differentiation of keratinocytes [13]. However, the most prominent, long-term radiation-induced side effects of the skin are telangiectasia and fibrosis [12,14]. Clinically, patients with dermal fibrosis show signs of increased stiffness and thickening of the affected skin [15]. Mechanistically, the development of fibrosis results from an interplay of cytokines and different cell types. The early inflammatory phase is characterized by an infiltration of immune cells, which initiates the restoration of the damaged tissue [13]. In the subsequent proliferative phase, infiltrating macrophages induce a trans-differentiation of stromal fibroblasts into myofibroblasts by secretion of the transforming growth factor beta (TGFβ) [15]. Myofibroblasts proliferate and secrete excessive collagen fibers and other extracellular matrix components (ECM) [15,16]. The ECM deposition is maintained over several years with a persistently elevated cytokine production [16]. Under normal circumstances of wound healing, the ECM expansion is provisional until the process of re-epithelialization occurs [17]. In the case of fibrosis, there is a pathological imbalance between synthesis and degradation of collagen [1]. Cytokine-driven ECM synthesis overbalances and culminates in fibrosis [16]. The main driver of the fibrotic process is the cytokine TGFβ [1], which is produced by different cell types, such as macrophages [15] or myofibroblasts [6]. TGFβ can be directly activated by ionizing radiation through dissociation of the latency-associated peptide [1]. TGFβ also plays an important role in activation, proliferation and differentiation of epithelial cells and fibroblasts into collagen-producing myofibroblasts [18]. In addition to a local expression of TGFβ at the exposed site, inflammatory cytokines are also systemically elevated after radiation treatment [19]. Cytokines that are highly concentrated in the plasma are able to induce radiation-related adverse effects in out-of-field organs. A systemic immune response activates immune cells in distant organs resulting in oxidative stress which can induce DNA damages and apoptosis in out-of-field normal tissue cells [19].

Side effects of the skin are observed in 90% of patients receiving radiation therapy [20]. Due to its clinical importance different scoring systems of acute radiation-induced side effects of the skin were developed. The most relevant scoring systems were introduced by the National Institutes of Health Common Toxicity Criteria-Adverse Event (CTCAE) and the Radiation Therapy Oncology Group (RTOG). Theses scoring systems assign radiation-induced side effects such as erythema and desquamation into four different severity grades [14,21]. A similar scoring system exists also for late radiation-induced morbidity (RTOG/EORTC) [21]. The scoring systems differ in the increments of the assigned grades [14]. For animal trials, the scoring systems are based on similar criteria as for humans, e.g., acute side effects in the skin such as erythema and desquamation can be assessed by different grades from no reaction to severe reaction in a mouse ear model [22,23]. However, a gold standard for the clinical rating of the severity of side effects in humans and animals still does not exist [14]. Therefore, different scoring systems have been used in clinical and experimental trials which hamper comparability between different studies. The comparability is also limited by the subjective assessment of radiation-induced toxicities by scoring systems, which deliver no quantitative data. It has been shown in radiobiological experiments using mice that the ear thickness increases if acute skin damage occurs due to X-ray irradiation. The measurement of the ear thickness might be an objective criterion for acute radiodermatitis [22]. In addition to these previously mentioned limitations of conventional scoring systems for humans and experimental animals, the relationship between acute and late side effects is not well understood. There are only a few studies suggesting a correlation between both lung function [24] and TGFβ1 expression [25], as acute reactions, correlate with pulmonary fibrosis as a late reaction. In the skin, a correlation between the number of atrophied hair follicles and the number of radiation-induced tumors was detected [24]. Clinical studies observed a relationship between acute and late toxicity in the gastrointestinal tract and in the skin after radiotherapy of prostate cancer [26] and breast cancer patients [27], respectively. In addition to that, a good correlation between acute and late skin effects was observed after fractionation in several mouse studies, e.g., desquamation (acute reaction) and necrosis (late reaction) [27]. Nevertheless, experimental data about a mechanistic relationship between acute and late side effects, especially in the skin, are rare despite the clinical importance of the skin, which is always affected by radiation because it is located at the beam entrance regardless of which tumor is irradiated [28].

Therefore, the aim of our study was to determine a correlation between acute and late side effects of the skin after a hypofractionated schedule. Doses up to 30 Gy per fraction were delivered daily to the skin for four consecutive days using a standardized mouse ear model, which allowed for quantification of acute and late radiation-induced side effects in the same animal model. Acute (ear thickness, erythema, and desquamation) and late side effects (fibrosis, chronic inflammation), and the abundance of TGFβ1-expressing cells in the skin were assessed up to 92 days following the first fraction. Our results indicate a dose-dependent positive correlation of the severity of acute side effects with a maximum reaction around day 15 after the first fraction. It also marks ear thickness as a quantitative scoring criterion for acute radiation-induced side effects, which is able to predict late tissue reactions in the mouse ear model.

## 2. Results

### 2.1. Analysis of Erythema and Desquamation

**Figure 1:**
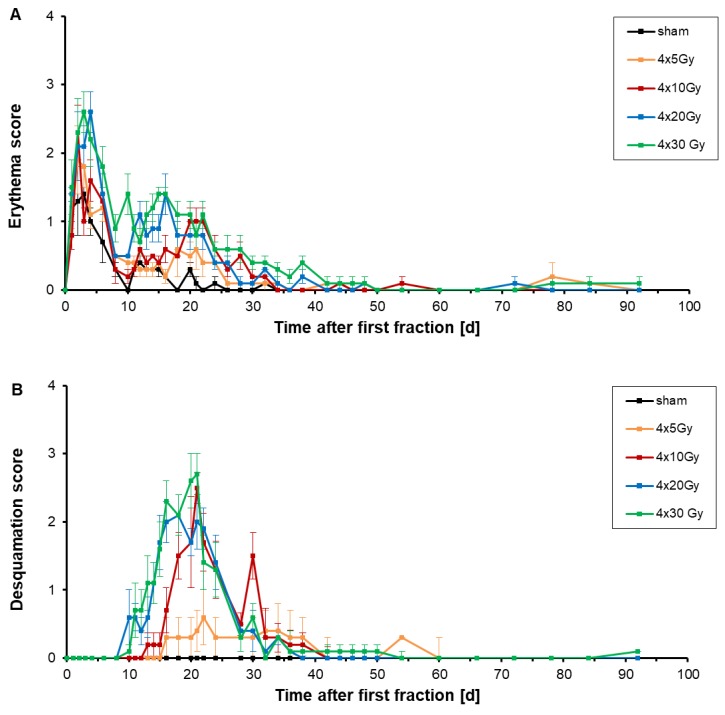
Semi-quantitative erythema score (**A**) and desquamation score (**B**) were measured on irradiated ears of 6–7 mice per dose group according to the grading scheme in Table 3 during the follow-up period of 92 days after a hypofractionation. Murine ears were irradiated with fractional doses of 0 Gy (black), 5 Gy (orange), 10 Gy (red), 20 Gy (blue) and 30 Gy (green). The error bars represent the standard errors of the mean (SEM).

The acute side effects erythema and desquamation were assessed independently in two different scores, depicted in Figure 1, during the follow-up period of 92 days after a hypofractionation schedule. Mouse ears were irradiated with four fractions with a fraction interval of 24 h. Fraction 1 was given on day 0, fraction 2 to 4 on days 1, 2 and 3, respectively (Figure 10).

Already one day after delivery of the first fraction, the erythema score increased (Figure 1A). This increase of the erythema score was present in all dose groups, even for sham-irradiations. During fixation of the ears in the mouse holder, ears were gently compressed which induced a flush of the skin. After fraction 2, 3 and 4 a further increase of the erythema score was measured which was more prominent for doses of 20 Gy and 30 Gy per fraction. This increase might be due to the combined effect of squashing and radiation, or longer fixation times due to longer irradiations for higher fractional doses. After completing irradiation (day 5–6), a prompt decrease of erythema was observed. The desquamation score remained unchanged during the time of irradiation.

On day 4 (first day after hypofractionated irradiation), the erythema score culminated at 2.2 ± 0.4, 2.6 ± 0.3, 1.6 ± 0.3 and 1.1 ± 0.2 for fractional doses of 30 Gy, 20 Gy, 10 Gy and 5 Gy, respectively. Sham-irradiated ears also showed a flush of the skin with a score of 1.0 ± 0.2 on day 4, likely induced by the mechanical fixation of the ears. On day 6 the erythema score dropped down to 1.8 ± 0.3, 1.4 ± 0.3, 1.3 ± 0.2, 1.2 ± 0.2 and 0.7 ± 0.3 on day 6 after four fractional doses of 30 Gy, 20 Gy, 10 Gy, 5 Gy and 0 Gy, respectively. A second increase of the erythema score appeared on day 15–16 with a score of 1.4 ± 0.1 and 1.4 ± 0.3 after both 4 × 30 Gy and 4 × 20 Gy (Table 1). After 4 × 10 Gy and 4 × 5 Gy irradiations, an erythema score of 1.0 ± 0.2 and 0.6 ± 0.4 was seen with score maxima between day 20–22 and day 18, respectively. After sham-irradiation, the erythema score of sham-irradiated ears culminated with a score of 0.4 ± 0.1 on day 12 and further decreased to 0.0 ± 0.0 on day 22. After 4 × 5, 4 × 10 and 4 × 20 Gy treatments, the erythema score decreased slowly to 0.0 ± 0.1, measured on day 34. On day 50, erythema also disappeared on 4 × 20 Gy and 4 × 30 Gy irradiated ears.

An increase of the desquamation score appeared initially 10 days after the first fraction of either 20 Gy or 30 Gy (Figure 1B). The start of the increase of the desquamation score was delayed when lower doses were delivered, e.g., 13 days after the first fraction of 10 Gy. Using doses of 30 Gy, 20 Gy, 10 Gy and 5 Gy per fraction the maximum score of desquamation was measured 21 days, 18 days, 21 days and 22 days upon the first fraction, respectively (Table 1). The observed desquamation was always dry and never developed into a moist desquamation.

As a conclusion, erythema is dose-dependent: the higher the dose per fraction the more pronounced and earlier the erythema develops. The severity of desquamation is apparently different between lower fractional doses of 5 Gy and fractional doses higher than 10 Gy, but the difference in severity between the high fractional doses is marginal. The evaluation of erythema and desquamation is however subjective and requires a standardized scoring system. Therefore, it is not suitable for a reliable and comparable grading of acute toxicities.

### 2.2. Analysis of Ear Thickness

**Figure 2:**
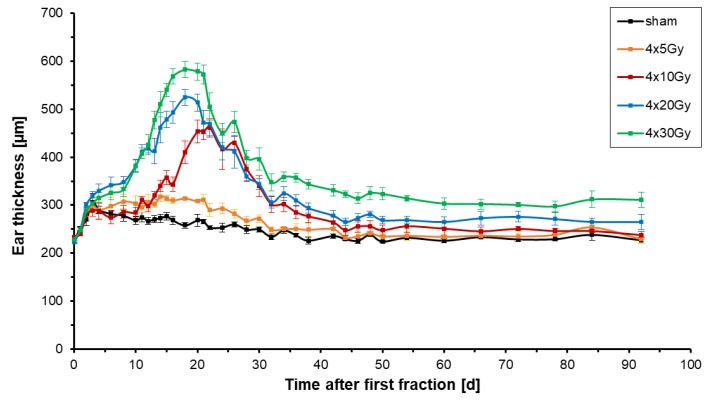
Thickness of irradiated right ears of mice after hypofractionated X-ray irradiation with a total of 4 fractions given every 24 h. Doses of 0 Gy (black), 5 Gy (orange), 10 Gy (red), 20 Gy (blue) or 30 Gy (green) per fraction were used per fraction. The plot shows the mean values for every dose group comprising 6–7 mice. The thickness of the irradiated ears was monitored for 92 days after the first fraction. The errors are given as the standard errors of the mean (SEM).

Acute intumescent inflammatory alterations (edema, vascular congestion, hyperemia) were assessed following hypofractionated X-ray radiation therapy by measuring the thickness of irradiated ears. Figure 2 shows the mean changes of the thickness of the right ears of 6–7 mice per dose group receiving four fractions with doses ranging from 0 Gy to 30 Gy per fraction.

The left ear of each mouse served as a control and had a constant thickness of 216.9 ± 6.1 µm. In all treatment groups, all mice had a similar initial thickness of the right ear of 227.6 ± 3.7 µm (Figure 2). With the start of treatment, the mean thickness of sham-irradiated ears increased slightly by around 80 ± 18 µm on day 3 but then dropped down again on day 4 once sham-irradiation was completed. The ears were gently compressed during the fixation in the mouse holder. The repetition of the mechanical stress on every day of irradiation caused a culmination of ear thickness to a maximum on day 3 (last day of irradiation). During the follow-up period of 92 days sham-irradiated ears reached a maximum thickness of 276.7 ± 15 µm on day 15 (Table 2) and returned to their initial thickness of around 230 µm on day 30 which remained constant from that day on.

All doses used in the X-ray fractions induced an increase of thickness of the right ear. After the first minor rise of ear thickness due to mechanical stress the ear thickness peaked 18 days after both 4 × 30 Gy and 4 × 20 Gy, and 22 days after 4 × 10 Gy (Table 2). After four fractions of 5 Gy, the ear thickness reached a plateau from day 14 onwards. Hypofractionation with four fractions of 30 Gy, 20 Gy, 10 Gy and 5 Gy led to an increase of ear thickness of 2.5, 2.4, 2.0 and 1.4 times the initial thickness, respectively. During the follow-up period, the thickness of irradiated ears decreased slowly to 250 ± 9.8 µm for 4 × 5 Gy, 250 ± 3.5 µm for 4 × 10 Gy, 265 ± 7.4 µm for 4 × 20 Gy, and 310 ± 17.1 µm for 4 × 30 Gy on day 92. The ear thickness never returned to the initial thickness, if four fractional doses of 20 Gy or 30 Gy were applied, demonstrating chronic ear swelling as part of chronic inflammation.

For all irradiation groups, it was observable that the temporal course of the radiation response was shifted to earlier time points for higher doses per fraction; the higher the dose per fraction the earlier the maximum ear thickness was reached and the stronger the ear swelling was present. In conclusion, ear thickness is a direct measurement of acute responses to radiation.

### 2.3. Dose-Dependency of the Maximum Ear Thickness, the Maximum Erythema and Desquamation Score

**Figure 3:**
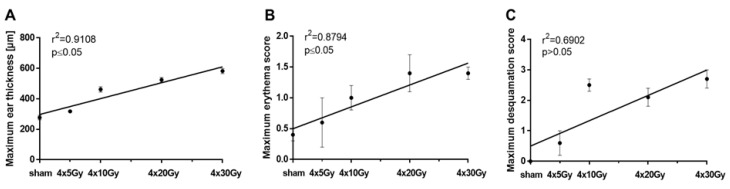
Comparison between the maximum ear thickness (**A**), the maximum erythema score (**B**) or the maximum desquamation score (**C**), and dose of irradiated ears after a 4-fraction course given one fraction per day. The days and the values on which the maximum erythema score, desquamation score and ear thickness were assessed are shown in Table 1 and Table 2. Correlations were calculated using Spearman’s correlation with linear regression. Significance levels are indicated. The errors are given as the standard errors of the mean (SEM).

In Figure 3A, the maximum ear thickness is plotted against the dose per fraction. It depicts that the maximum swelling correlated linearly (y = 10.42*x + 297.2) with a significance of *p* ≤ 0.05 with the dose per fraction after a hypofractionated radiation treatment. Figure 3B shows that the maximum erythema score significantly (*p* ≤ 0.05) correlated with the dose of all used hypofractionation schedules (y = 0.04*x + 0.49). Whereas there was no correlation between the maximum desquamation score and the doses per fraction (*p* > 0.05, Figure 3C).

The higher the dose per fraction the thicker is the irradiated ear. The maximum thickness of irradiated ears is a dose-dependent measurement.

### 2.4. Comparison of the Maximum Acute Reaction Score and the Maximum Ear Thickness between Hypofractionated and Single X-ray Dose Irradiation

**Figure 4:**
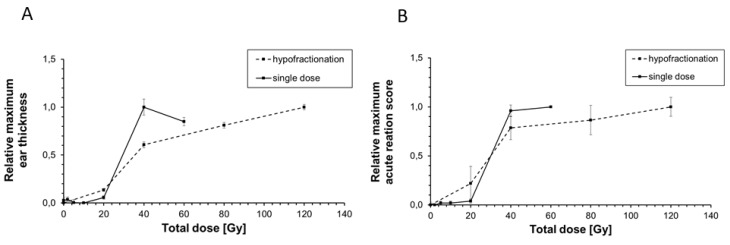
Comparison of the relative maximum ear thickness (**A**) and relative maximum acute reaction score (**B**) of murine ears after irradiation with both single (solid line) and hypofractionated (dashed line) X-ray doses. The maximum measured ear thickness (Table 2) and the maximum acute reaction score (Table 1) were set to 1, the minimal ones to 0 in order to calculate the relative values. For single dose irradiation, the maximum ear thickness and the maximum acute reaction score were used from Girst et al., 2016 [22] using X-ray doses of 0 Gy, 2 Gy, 5 Gy, 10 Gy, 20 Gy, 40 Gy and 60 Gy. For hypofractionation, the maximum erythema score and the maximum desquamation score (Figure 1) were summed up to an acute reaction score. The total doses of 20 Gy, 40 Gy, 80 Gy and 120 Gy correspond to the used four fractional doses of 5 Gy, 10 Gy, 20 Gy and 30 Gy, respectively. The errors are given as the standard errors of the mean (SEM).

In a previously performed study by Girst et al., 2016 [22], ear thickness, erythema and desquamation as acute side effects were assessed after single dose X-ray irradiation of murine ears. Erythema score and desquamation score were combined to one acute reaction score (see Section 4.3). Here, we want to compare the severity of both ear thickness (Figure 4A), and the acute reaction score (Figure 4B) between hypofractionated and single dose irradiation. In Figure 4, relative values are illustrated in order to reduce a difference in the individual assessment of acute side effects.

Figure 4A shows that a single dose of 40 Gy (1 × 40 Gy) induced a relative maximum ear thickness of 1.0 ± 0.08 while 4 × 10 Gy which corresponds to a total dose of 40 Gy induced a lower relative maximum ear thickness of 0.61 ± 0.03. After 4 × 10 Gy a similar reduction of the maximum acute reaction score was measured compared to that after 1 × 40 Gy (0.78 ± 0.1 vs. 0.96 ± 0.06, Figure 4B). In contrast to that, there was only a marginal difference of the maximum ear thickness of about 0.08 between 4 × 5 Gy (total dose of 20 Gy) and 1 × 20 Gy despite the same total dose was delivered. At higher doses, the delivering of 4 × 20 Gy (total dose of 80 Gy) to murine ear resulted in an ear thickness of 0.81 ± 0.03 and acute reaction score of 0.87 ± 0.15. The same maximum ear thickness of 0.85 ± 0.04 was measured after 1 × 60 Gy despite 20 Gy less was applied. A similar maximum acute reaction score of 1.0 was observed for single doses higher than 40 Gy (0.96 ± 0.06) and total doses of 120 Gy (4 × 30 Gy, 1.0 ± 0.09) indicating that a much higher dose is necessary to observe both the same maximum ear thickness and acute reaction score in the murine ear skin if hypofractionation is used.

### 2.5. Histopathological Analyses of Irradiated Ears

**Figure 5:**
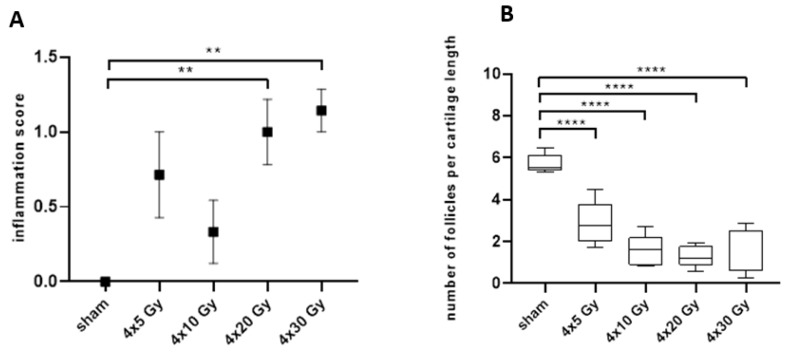
Semi-quantitative analysis of the inflammation score (**A**) and quantification of hair follicle profiles within pre-defined region of interest. (**B**) in histological sections of irradiated ears on day 92 after sham-irradiation or a 4-fraction course with doses of 5 Gy, 10 Gy, 20 Gy and 30 Gy per fraction. One-way ANOVA with Kruskal-Wallis test and Dunn’s post hoc test was used for statistical analysis. Asterisks indicate significant differences: ** *p* ≤ 0.01, **** *p* ≤ 0.0001.

In addition to the assessment of acute side effects in the skin, late irradiation-induced side effects were also examined by qualitative and quantitative histopathological analyses. In sections of the irradiated ears, the infiltration of inflammatory cells as a late radiation-induced side effect was acquired 92 days post-irradiation, using a semi-quantitative grading scheme (see Section 4.3). Figure 5A shows an increase of the inflammation score with increasing dose 92 days after hypofractionated radiotherapy. The frequency (i.e., number of affected animals) and severity of inflammatory lesions (grade 0 to 2) continuously increased in groups with increasing radiation doses. At day 92, the severity of inflammatory lesions significantly increased after four fractional doses of 20 Gy or 30 Gy demonstrating that a chronic inflammatory reaction has been developed.

The loss of hair follicles represents an additional, side effect of radiation [11]. In Figure 5B the number of section profiles of hair follicles within a defined region of interest (ROI) is displayed on day 92. There was a visible trend that the number of follicle profiles per ear section length decreased as the fractional dose increased. Already 4 × 5 Gy resulted in a highly significant loss of follicle profiles per ROI. With increasing dose further from 4 × 10 to 4 × 30 Gy, the number of section profiles of hair follicles was further reduced, and follicle profiles were almost completely absent. The absence of hair follicle profiles at a late time point (e.g., day 92) indicates that the radiation-induced damage of hair follicles does not recover probably due to complete sterilization of stem cells in the hair bulb. 

**Figure 6:**
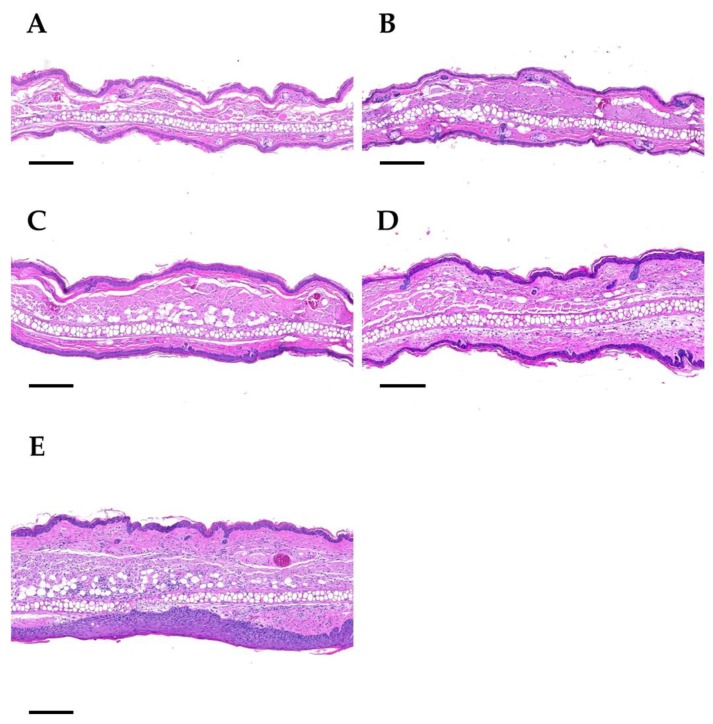
Representative histological images of ears on day 92 upon a 4-fraction course with different doses per fraction: sham (**A**), 5 Gy (**B**), 10 Gy (**C**), 20 Gy (**D**), 30 Gy (**E**). Panel **A**: Sham-irradiated. The central cartilage of the pinna is flanked by a layer of skeletal muscle, a dermal layer of moderate thickness and the epidermis. Hair follicles, nerves, sebaceous glands, and blood vessels are present. Panel **B**: 4 × 5 Gy. The dermal layer of the ear skin is slightly thickened. Hair follicles are rarely present. Panel **C**: 4 × 10 Gy. The dermal layer is further increased, and the epidermis is mildly hyperplastic. Hair follicle profiles are almost completely absent. No sebaceous glands are present. Panel **D**: 4 × 20 Gy. The dermal layer of the skin is markedly thickened and infiltrated by a mixed population of inflammatory cells. No hair follicles and sebaceous glands are detectable. Panel **E**: 4 × 30 Gy. The ear section displays severe dermal thickening and inflammatory cell infiltration, as well as marked epithelial hyperplasia. No hair follicles and sebaceous gland section profiles are present. Paraffin sections were stained with hematoxylin and eosin. Scale bar: 200 µm.

Pronounced epidermal hyperplasia was regularly present in ear sections after four doses of 30 Gy (Figure 6). While in low dose groups the thickness of the epidermal layer is comparable to sham-irradiated ears, an up to two-fold increase in dermal thickness was observed after 4 × 30 Gy. As a conclusion, after high doses (>4 × 10 Gy) chronic side effects such as inflammation and epidermal hyperplasia are induced. 

### 2.6. Correlation of Acute and Late Side Effects after Hypofractionation

The results in the previous Section 2.2 and Section 2.5 showed a strong increase of the dermal thickness of ears especially after receiving 4 × 30 Gy. Therefore, we analyzed the extent of dermal fibrosis, as well as the abundance of TGFβ1-expressing cells in irradiated ear sections. 

**Figure 7:**
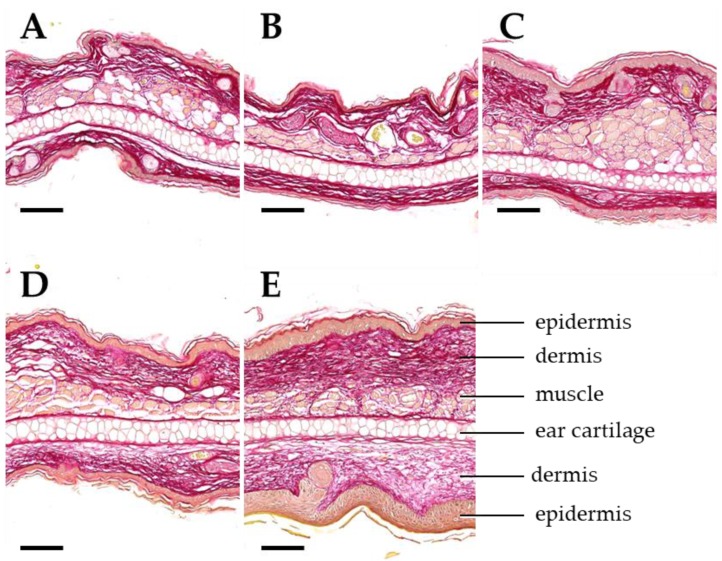
Dose-dependent development of dermal fibrosis. Representative images of ear sections on day 92 after a 4-fraction course using different doses per fraction: sham (**A**), 5 Gy (**B**), 10 Gy (**C**), 20 Gy (**D**), 30 Gy (**E**). Sections are stained with Sirius red for demonstration of collagenous connective tissue (dark red color). Important anatomical structures are indicated in **E**. Ear thickness and connective tissue deposition in the dermal layers of the ear skin increase with the radiation dose. Paraffin sections. Scale bar: 100 µm.

Sirius red staining was used to visualize collagen in ear section on day 92 (Figure 7). Parallel to the increase of the ear thickness, the deposition of collagen fibers was increased after 4 × 10 Gy and 4 × 20 Gy. If 4 × 30 Gy was applied, the collagen deposition is more pronounced contributing to a two-fold increase of the ear thickness. Due to this observation, we wanted to analyze the correlation between acute side effects indicated as ear thickness, and late side effects such as fibrosis, the number of TGFβ1-expressing cells and chronic inflammation.

**Figure 8:**
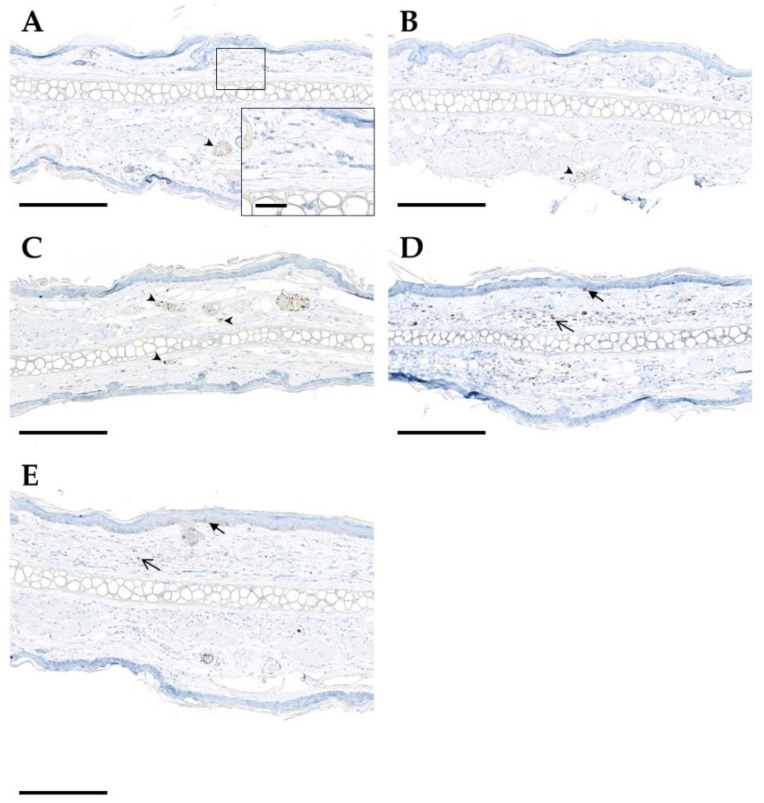
Immunohistochemical detection of TGFβ1 (brown color) in mouse ear sections on day 92 following hypofractionation with different doses per fraction: sham (**A**), 5 Gy (**B**), 10 Gy (**C**), 20 Gy (**D**), 30 Gy (**E**). Positive endothelial and intravascular inflammatory cells are indicated by arrowheads, epidermal immunoreactivity by closed arrows, and immunoreactivity of dermal cells by open arrows. Paraffin sections of murine ears are shown. Chromogenic substrate: diaminobenzidine (DAB), nuclear counterstain: hematoxylin. Scale bar: 200 µm (for zoom-in image 20 µm).

Quantification of the number of TGFβ1-expressing cells in histological ear sections on day 92 after hypofractionation showed that TGFβ1 was mainly expressed by endothelial cells, intravascular immune cells as well as epidermal cells of the basal layer and dermal spindle cells (Figure 8). With increasing dose, the number of TGFβ1-postive cell profiles slightly increased.

**Figure 9:**
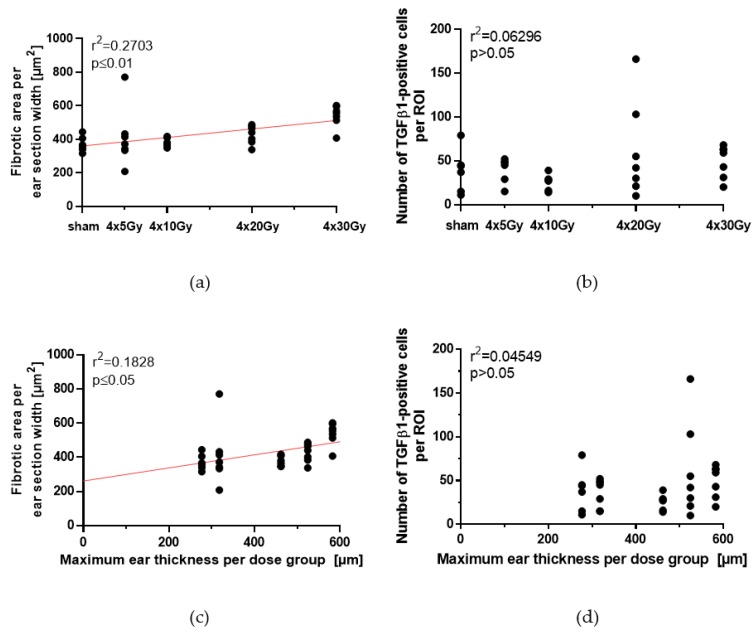

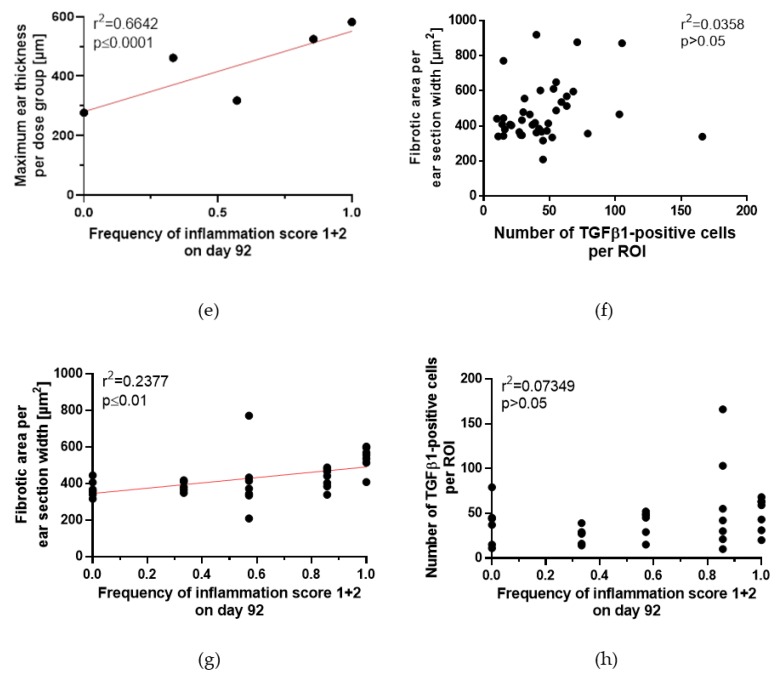
Quantification of the fibrotic area and the abundance of TGFβ1-expressing cells in ear sections on day 92 following a 4-fraction course with different doses per fraction. Collagen was stained by Sirius red and TGFβ1-expressing cells were identified by immunohistochemistry and quantified within the pre-defined ROI, using automated digital image analysis. (**a**) Correlation of the fibrotic area on day 92 with the dose per fraction. (**b**) Correlation of the number of TGFβ1-expressing cells on day 92 with the dose per fraction. (**c**) Correlation of the fibrotic area with the maximum ear thickness during the acute reaction (see Section 2.2, Table 2). (**d**) Correlation of the number of TGFβ1-expressing cells on day 92 with the maximum ear thickness during the acute reaction (see Section 2.2, Table 2). (**e**) Correlation between the maximum ear thickness as acute reaction (see Section 2.2, Table 2) and the frequency of both inflammation score 1 and 2 in all animals on day 92. (**f**) Correlation between fibrotic area and number of TGFβ1-postive cells. (**g**) Correlation between fibrotic area and the frequency of both inflammation score 1 and 2 in all animals on day 92. (**h**) Correlation between number of TGFβ1-expressing cells and the frequency of both inflammation score 1 and 2 in all animals on day 92. Correlations were calculated using Spearman’s correlation with linear regression. Significance *p*-values are indicated.

Collagen deposition in Sirius red stained ear sections was quantified as the fibrotic area per mm section length, using automated digital imaging analysis. The dose-dependency of both the fibrotic area and the number of TGFβ1-positive cells per ROI is depicted in Figure 9a,b. With increasing dose per fraction, the fibrotic area per mm section width increases, but not the number of TGFβ1-positive cells per ROI. The applied dose per fraction correlated significantly with fibrotic area (*p* ≤ 0.01), while there was no correlation with TGFβ1 expression in irradiated ears (*p* > 0.05).

Mouse ears irradiated with 5 Gy per fraction had a maximum thickness of 317.9 ± 8.2 µm (see Table 2) and a fibrotic area of 411.1 ± 174.8 µm^2^ and 36 ± 16 TGFβ1-positive cells per ROI. In mouse ears irradiated with a higher dose of 30 Gy per fraction with a maximum ear thickness of 582.4 ± 16.9 µm (Table 2) showed a fibrotic area of 520.2 ± 66.2 µm^2^ and 50 ± 18 TGFβ1-positive cells per ROI. Therefore, the size of fibrotic area in the skin is correlated with the maximum ear thickness (*p* ≤ 0.05), which is observed after irradiation using fractional doses from 0 Gy to 30 Gy (Figure 9c). However, there was no correlation between the number of TGFβ1-positive cells and the maximum ear thickness (*p* > 0.05, Figure 9d). Moreover, the maximum ear thickness correlated with the inflammation score (Figure 9e), e.g., no inflammation was found in sham-irradiated ears with a thickness of 276.7 ± 15 µm (Table 2), but 100% of 4 × 30 Gy irradiated ears with a thickness of 582.4 ± 16.9 µm showed an inflammation score of 1 and 2.

Since different types of inflammatory cells express TGFβ1, it was of interest to evaluate, if there was a correlation between the number of TGFβ1-expressing cells, fibrosis and inflammation on day 92 after hypofractionation. Our data show no correlation between the severity of fibrosis and the number of TGFβ1-expressing immune cells present in the examined sections at the given time point (*p* > 0.05, Figure 9f), whereas there was a correlation between the fibrotic area and inflammation (*p* ≤ 0.01, Figure 9g). The number of TGFβ1-expressing cells was not correlated to the severity of inflammation (Figure 9h). In summary, there are significant correlations between the thickness of ears as an acute reaction, and fibrosis and inflammation score as late side effect on day 92.

## 3. Discussion

The use of different semi-quantitative scoring systems for acute and late side effects in patients and experimental animals does not allow for a reliable comparison between different studies. Moreover, there is no scoring criterion which allows for the prediction of the chance for occurrence and severity of late, possibly chronic side effects. The highest risk to develop side effects is in the skin, which is always irradiated during the treatment of deep-seated tumors. Therefore, in the present study, we examined the acute and late side effects of murine ears after a hypofractionation schedule with four daily fractions using doses from 0 Gy to 30 Gy per fraction. Our aim was to figure out whether the evaluation of acute side effects is able to predict the occurrence of late side effects after hypofractionation.

Our study demonstrates that the severity of the acute side effects, erythema and ear thickness, are significantly dose-dependent. Moreover, in contrast to erythema and desquamation, ear swelling represents a direct pendant of acute responses (edema, vascular congestion, hyperemia) to ionizing irradiation, and can adequately be quantified by measurement with a caliper. Therefore, ear swelling is suitable as a quantitative scoring criterion for acute side effects. Our study showed that quantitatively determined ear swelling as an acute side effect is significantly correlated with the fibrotic area and inflammation on day 92 after radiation exposure. Therefore, measuring the ear thickness can predict the development of late side effects in the skin at late time points after radiation treatment in the mouse ear model.

We quantified the area of deposited collagen in the ear sections as marker for fibrosis. Fibrosis correlated significantly with the applied dose which agrees to the result of another previously published study [15]. In the mouse ear model, fibrosis was quantified on day 92 (13 weeks post-irradiation). Comparable studies observed dermal fibrosis in mice at week 21 after irradiation with 35 Gy [29] or 12 weeks post-irradiation with doses up to 25 Gy of gamma-rays [30]. The differences between time points for measuring of fibrosis could be explained by the use of different mouse strains and different irradiated parts of the body, all differently prone to radiation-induced toxicities. These mouse studies delivered single doses in contrast to our hypofractionated schedule which is presumed to reduce toxicities. These results are well in line with our study in which no increased collagen deposition was observed in the mouse ear on day 92 after a dose up to 4 × 20 Gy.

In addition to fibrosis, we quantified the number of TGFβ1-expressing cells in ear sections. Our study does not show a correlation between ear swelling and the number of TGFβ1-expressing cells per section ROI. However, there was a slight, but not significant increase in the cellular expression of TGFβ1 but it was only detectable after 4 × 30 Gy. Furthermore, the time point (92 days) for the quantification of TGFβ1 may have been too early since fibrosis develops gradually after radiation exposure. Fibrosis represents the final result of a chronic inflammatory reaction [31]. There are three distinct increases of TGFβ1 expression in murine skin [32]. After a first and a second wave at day 1–2 and day 14–28 post-irradiation, a third peak was observed after 9 months starting to elevate after three months [32]. We can infer from the study mentioned earlier that both a higher TGFβ1 expression and thus higher collagen deposition might be detectable later than three months after hypofractionation. These findings are in agreement with the observed chronic inflammation (increase of the inflammation score) in murine ears on day 92 after hypofractionation. These increase number of inflammatory cell types might stimulate the development of fibrosis. Since fibrosis develops gradually over a prolonged period of several months after radiation therapy, we will quantify the fibrotic area and the tissue expression of TGFβ1 at later time points after hypofractionation in a future experiment, also using quantitative RNA- and protein analysis.

Our study focused on the locally increased expression of TGFβ1 in the ear. However the role of systemically increased TGFβ1 cannot be neglected. It could be that TGFβ1 circulating in the plasma of irradiated mice may activate immune cells in out-of-field organs as shown in previous studies by Ventura et al., 2017 [19] and activated inflammatory cells may promote oxidative stress, DNA damage and apoptosis in distant normal cells and in turn, it can also induce fibrosis and local inflammation in distant organs [19]. However, the systemic response of radiation exposure interplays with the genetic background of the host [33]. Therefore, it needs to be elucidated whether TGFβ1 is systemically increased and if it plays a distant role in the mouse ear model. 

In addition to late side effects, we also evaluated the time course and the dose-dependency of acute side effects. It has been shown that erythema, as a cardinal symptom of acute inflammatory reactions, manifests as one of the first pathological signs of radiation [34]. Our data shows that the erythema score is already elevated after the first fraction. This early erythema might also be caused by the mechanical stress during ear fixation. However, the erythema score was still high at day 5 when the treatment was completed and consequently no daily exposure to mechanical stress was present anymore. A similar increase of the score of dorsal skin lesions with different maximum intensities was observed at day 7 after a single dose exposure of 20 Gy, 40 Gy and 80 Gy [35] which confirms the early onset of erythema and also the measured significant dose-dependency of the severity of erythema in murine ears. The increase of erythema score persisted for about two weeks for each dose. During this period, the desquamation score also starts to rise with increasing dose (not significant). Desquamation is a later acute pathological sign of radiation damage [14]. With an increasing dose, the advent of the desquamation reaction occurred later; it started to increase at day 10 after both 4 × 30 Gy and 4 × 20 Gy, at day 13 after 4 × 10 Gy and at day 16 after 4 × 5 Gy. A significant dose-dependent increase of the desquamation score was not detected in our study. Nevertheless, an increase dose-dependent progression of skin lesions was found in other mouse studies, observing desquamation at about day 14 after single dose exposure of 20 Gy, 40 Gy and 80 Gy [35] confirming the results of our study. Desquamation was resolved at around day 30 after doses below 4 × 20 Gy as also shown in the mouse skin after irradiation with single doses of 20 Gy [22] and 30 Gy [36]. 

After hypofractionation, an increase of ear thickness was observed which culminated on day 18 with a thickness of about 600 µm after 4 × 30 Gy. This ear thickness is much lower compared to X-ray single dose irradiations in the same mouse ear model showing a maximum ear thickness of 900 µm around day 25 after either 40 Gy or 60 Gy single doses [22]. When comparing the total doses which were delivered to murine ears during single dose and hypofractionated irradiation, a total dose below 20 Gy induced a similar increase in ear thickness and acute reaction score independent of the fractionation schedule. However, an increased sparing of the skin was observed at higher doses, e.g., a total dose of 40 Gy induced a less pronounced ear swelling and acute reaction score when X-rays were applied as a hypofractionated dose. To detect the same ear thickness or the same acute reaction score, a much higher X-ray dose can be delivered if murine ears are fractionally irradiated. The comparison of the ear thicknesses between both experiments should be done with caution. A difference in the individual scoring between several examiners is assumed. Furthermore, the assessment of erythema and desquamation constitutes a more subjective criterion than the measurement of the ear thickness.

The macroscopic increase of the ear thickness in the mouse ear model can be attributed, partly, to an increase of the thickness of the epidermis [37,38]. Our study shows a long-lasting hyperplasia of keratinocytes after 4 × 30 Gy. These results can be confirmed by a hind limb skin model which observed an apparent thickening of the murine epithelium after a single dose exposure of 30 Gy and 40 Gy [39]. A second, major contributor to the prompt increase of ear thickness appears to be the accumulation of inflammatory cells upon irradiation as shown in radiation dermatitis studies [37]. The inflammatory phase is the first step of cutaneous wound healing starting within the first three days post-irradiation [20] leading to the continuous increase of ear thickness from day 5 in our study. Immune cells are recruited by pro-inflammatory mediators (e.g., interleukin 17C) expressed by keratinocytes upon X-ray exposure [37]. These mediators may also trigger the hyperproliferation of keratinocytes by an autocrine feedback loop [37] which induces the cellular regeneration of the wound during the second phase of healing [40]. This process is linked to the hyperplasia of keratinocytes. In a pig skin model, healing of the skin is initially associated with a first decrease of basal cell density followed by an increase on day 14–22 after 15 Gy single dose exposure, or even later between days 25–35 after single exposure of 20 Gy [38] which agrees to the beginning of cellular regeneration on day 10–14 in humans [14]. In contrast to that, we observed an increase of the ear thickness between the days 18–22 (depending on the dose). This observation suggests that cells, which were killed by radiation, were removed by inflammatory cells enabling the replacement of dead keratinocytes by new ones in order to maintain the skin barrier. However, in addition to increased repopulation, increased differentiation may also take place among the regenerated keratinocytes. Regarding the observed increase of ear thickness in our study, the infiltration of immune cells might compensate the decline of basal cell density, as shown in the pig skin model, suggesting that inflammation might be the one of the major contributors to acute radiation-induced ear thickening and a relevant contributor to the chronic ear thickening. Therefore, the ear thickness is a direct measurement of the healing process. Another aspect when comparing our data to the pig skin model would be the applied fractionated schedule. The first fraction destroys some basal keratinocytes followed by further fractions which destroy the remaining keratinocytes [14]. According to this, higher doses lead to an increased cell death and therefore to a reduced basal cell density. This suggests that there is a massive infiltration of immune cells at high doses corresponding to the observed increase of the maximum ear thickness with increasing dose. This proposed mechanism agrees with a study of Morris and Hopewell in 1988 [38] measuring the dependency between the severity of cell depletion, time of maximum depletion and dose. In line to our study, ear swelling, as a sign for cell damage and the healing process, correlates with both an increasing fractional dose and an increasing latency time: The higher the fractional dose, the earlier the ear swelling peaked and the higher is the severity of swelling. After fractional doses of 30 Gy or lower, ear swelling was completely resolved 45 days post-hypofractionation which is in agreement with the findings of a restored basal cell density at day 48 in the pig skin model [38].

## 4. Material and Methods

### 4.1. Ear Irradiation

Female BALB/c mice with an age of 8–12 weeks were obtained from Janvier (Janvier Labs, Saint-Berthevin Cedex, France). Mice were hosted at the experimental sites of the Helmholtz Zentrum München GmbH (Munich, Germany) according to the respective institutional guidelines and the German animal welfare regulations with permission of the local authorities (Regierung von Oberbayern, project license ROB-55.2-2532.Vet_02-17-9). The animals were kept at 20–24 °C, 45–65% relative humidity, at 12 h light-dark cycle and fed with commercial laboratory animal diet and water ad libitum.

**Figure 10:**
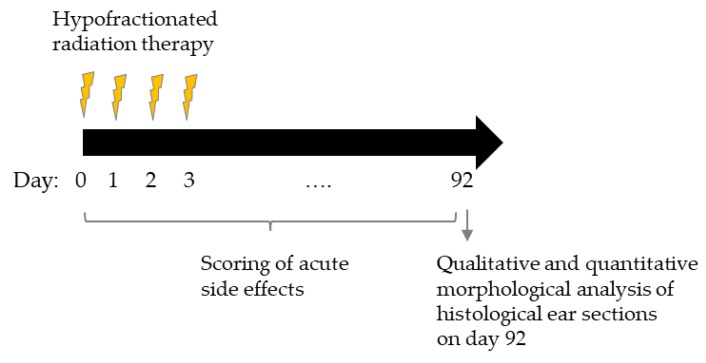
Schematic overview of the mouse ear study. Mouse ears were irradiated on 4 consecutive days with doses ranging from 0 Gy, 5 Gy, 10 Gy, 20 Gy to 30 Gy per fraction. Fraction 1 was given on day 0, fraction 2, 3, and 4 was given on day 1, 2 and 3, respectively. Acute side effects were assessed during irradiation and during a follow-up period. On day 92, sections of the mouse ears were assessed for examination of chronic side effects.

For irradiation, the animals were anaesthetized intraperitoneally with a mixture of 1 mg/mL Medetomidin, 5 mg/mL Midazolam and 0.05 mg/mL Fentanyl. Only the right ear of each mouse was irradiated with a total of four fractions delivered every 24 h. The left ear of each mouse served as an internal control. The dose per fraction was 5 Gy, 10 Gy, 20 Gy and 30 Gy using 70 kVp X-rays at the conventional X-ray cabinet RS225 (XStrahl Limited, Camberley, UK). A 3 mm-thick aluminum filter was used for beam hardening. The irradiation field had a size of 7.2 mm × 7.2 mm. 6–7 mice per dose group were irradiated and compared to sham-irradiated mice, which were treated according to the same protocol but without turning on the X-ray beam. In total, the right ears of 40 mice, including 34 irradiated and 6 unirradiated control animals, were used. Fraction 1 was given on day 0, fraction 2 to 4 on days 1, 2 and 3, respectively (Figure 10). Acute side effects were measured during a follow-up period of 92 days. Late side effects were assessed 92 days after the first fraction.

### 4.2. Ear Positioning and Accuracy

**Figure 11:**
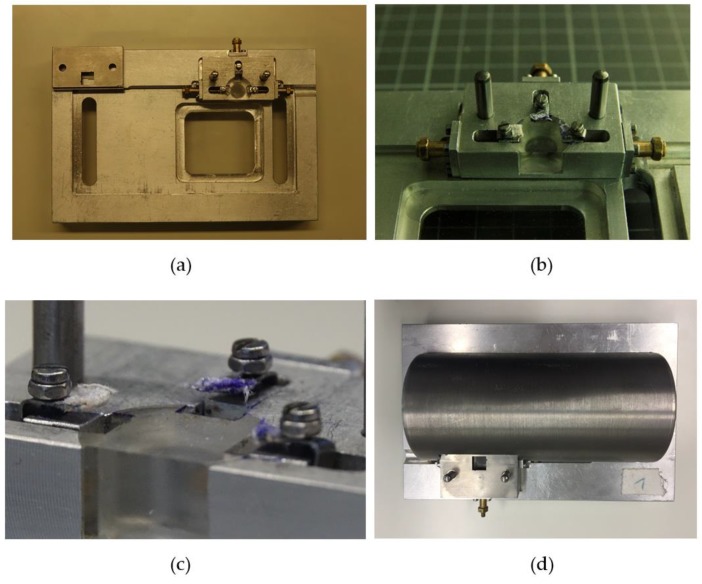
(**a**) Mouse ear holder for irradiation setup. (**b–c**) Three movable clamps can be seen directly next to the Plexiglas area on the top right corner with their corresponding golden millimeter screws. (**d**) The entire body of the mouse was shielded by a tungsten shield. Parts of the ear which were not irradiated were also shielded by a tungsten collimator.

For fractionated irradiation, the positioning of the mouse ear was essential. The field of the mouse ear, which was irradiated in fraction one should be reproduced for the following fractions as accurately as possible. For this purpose, a special mouse ear holder was developed (Figure 11) and supplementary software was used.

The holder of the ear consists of a lying surface with a Plexiglas window to observe the mouse during irradiation. A small stage with an embedded Plexiglas insert is mounted at the location of the head. In Figure 11b, the two big pins are used to fix the tungsten shield, which also defines the irradiation field. Three clamps surround the Plexiglas for ear positioning. The clamps were taped up to spare the ears as much as possible from mechanical stress (Figure 11c). By tightening the screws, the ear could be fixed and by turning the golden millimeter screws the clamps and thus the ear can be moved.

**Figure 12:**
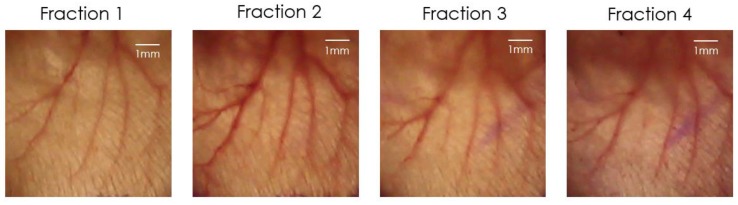
Illustration of the irradiation field for every fraction during the 4-fraction course.

For validation of the correct ear position und thereby of the irradiation field, a picture of the fixed ear was taken daily before the fraction was delivered. To perform the matching a Sony RX10 III camera (Sony, Tokio, Japan) was installed directly above the ear and was connected to the Sony software “imaging edge”. The software allows a live view of the current image, which enables the superposition of the live view with a template with a steplessly adjustable opacity. The template used was the image taken at day of the first fraction, which was meant to be reproduced for the following fractions. This image was superimposed with the current live view for the matching of the fractions 2, 3 and 4. By moving the ear with the millimeter screws the blood vessel structure could be adjusted until it was aligned with the image of fraction one. Using this procedure, an accuracy of around 250 µm was achieved. Figure 12 illustrates the field on the ear, which was irradiated on every day during the hypofractionation schedule.

### 4.3. Scoring of Acute Side Effect: Erythema, Desquamation and Ear Swelling

The soring endpoints were erythema, desquamation and ear thickness. The acute side effects of both the unirradiated left ear and the irradiated right ear were measured over a period of 92 days post-irradiation. All three endpoints were assessed before irradiation (day 0), between the fractions (day 1, 2, 3) and the day after the fourth fraction (day 4) followed by an assessment on every other second day. The scoring frequency was increased once either the ear thickness or acute side effects increased and decreased once the acute reactions decreased. The acute side effects were assessed during a follow-up period of 92 days.

The ear swelling was determined by measuring the ear thickness using a specially adapted electronic external measuring gauge (C1x079; Kröplin GmbH, Schlüchtern, Germany), with measuring contacts of 6 mm in diameter [22]. This caliper allows the measurement of ear thickness within the whole irradiation field without applying pressure to the ear and therefore without squashing of the ear.

Erythema and desquamation were assessed in four grades (Table 3). This system has been used in our laboratory in previous studies [22]. The scoring was always performed by the same two examiners. Scoring of each mouse was carried out blindly in order to limit individual differences in scoring. Both scores were summed up to one acute reaction score [22] for the comparison of radiation-induced side effects after hypofractionated and single dose irradiations (see Section 2.4).

### 4.4. Histopathological Analysis of Skin Toxicities on Day 92

**Figure 13:**
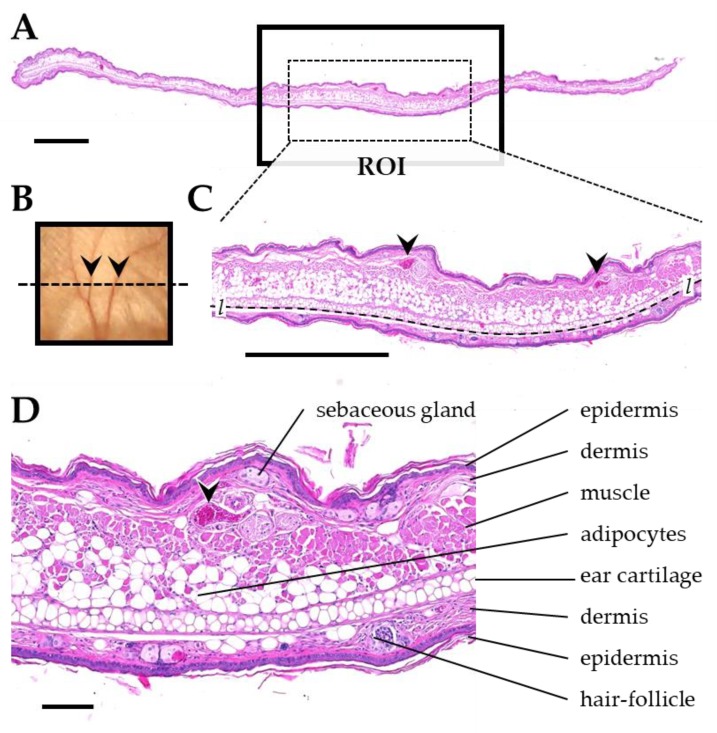
Anatomical landmarks used for definition of the ROI in transversal ear sections. (**A**) Histological section of the pinna. The radiated region of the pinna is indicated by a black box. The dotted line in (**B**) indicates the orientation of the section through the radiated area of the pinna. (**C**) ROI sampled for histological analysis and morphometric analysis (dotted rectangle in **A**). The ROI contains at least two section profiles of ear veins (arrowheads) and skeletal musculature. The dotted line marks the interface of the ear cartilage, used to measure the length *l* of the ROI (i.e., the section width). (**D**) Detail enlargement of the image shown in **C**, demonstrating relevant histological structures. Formalin-fixed paraffin embedded sections, H&E staining. Scale bar = 1 mm in **A** and **C**, and 100 µm in **D**.

After a follow-up period of about 92 days all animals were sacrificed. The excised ears (right) were fixed in 4% (*w/v*) neutrally buffered formaldehyde solution, sectioned and subsequently embedded in paraffin. Transversal ear sections from the center of the irradiation fields with 3 µm thickness were prepared for histological and immunohistochemical analyses. Histological sections were stained with eosin and hematoxylin, or Sirius red for quantification of fibrosis, respectively. For detection of TGFβ1, a monoclonal rabbit anti-mouse TGFβ1 antibody (abcam EPR21143, Abcam, Cambridge, UK) and an horseradish peroxidase (HRP)-coupled secondary antibody (goat anti rabbit IgG, BA-1000, Biozol Vector, Eching, Germany) were used. Appropriate positive (murine spleen) and negative controls were included in the analysis. Before the analysis and quantification of skin toxicities was performed, a region of interest (ROI) was defined in the center of the irradiated section area, using anatomical landmarks (Figure 13). This ROI had a width of about 3.5 mm and harbored skeletal musculature of the pinna and at least two section profiles of ear veins and/or ear nerves.

Inflammation within the section ROI was graded from 0 to 2 using a semi-quantitative grading scheme. Grade 0 shows no relevant infiltration of extravascular neutrophils, no vasodilation or edema. In grade 1, extravascular neutrophils and other mononuclear infiltrating cells appear scattered. Vasodilation and edema are present. Fibrosis may be present. Grade 2 is characterized by a multifocal to diffuse appearance of numerous extravascular neutrophils and other mononuclear infiltrating cells. Additionally, vasodilation and edema are present together with subepidermal fibrosis and epithelial thickening. Ear sections were assigned blindly to one of these three inflammation grades.

The quantification of fibrosis and TGFβ1-positive cells was performed by automated digital image analysis (Definiens Architect XD, Definiens Inc., Carlsbad, CA, USA).

### 4.5. Statistical Analyis

The statistical significance of the differences in the histological parameters measured for different doses per fraction was estimated using the program Graphpad Prism 8 (https://www.graphpad.com/scientific-software/prism/). The *p* values were calculated based on the variance analysis (one way-ANOVA test) with Kruskal-Wallis-Test and Dunn’s post hoc test. Correlations were performed using the Spearman’s correlation with linear regression.

## 5. Conclusions

In conclusion, our findings demonstrate that acute and late radiation-induced side effects of the skin are dose-dependent after a hypofractionated regime. We found that doses up to 20 Gy per fraction caused only reversible effects besides follicle loss while a higher fractional dose of 30 Gy indicates the start of chronic side effects such as fibrosis. Moreover, our results show that ear thickness as a quantitative measurement for ear swelling is able to predict chronic side effects such as fibrosis and inflammation in a mouse ear model. This scoring system can be transferred to other animal models in order to measure acute and late side effects in the ear. Therefore, the results of our in vivo study deliver a numerical scoring criterion of acute side effects after radiation for a better comparison between different treatment modalities like spatially fractionated radiation therapy or high linear energy transfer (LET) particle therapy in future. In future studies, the presence of TGFβ1 in the blood should be analyzed in order to better understand the systemic effects on distant organs.

## Figures and Tables

**Table 1:** Maximum erythema score and desquamation score of ears after receiving a 4-fraction course with different X-ray doses. The time after the first fraction is illustrated at which either the erythema or desquamation score was maximal. The errors are given as the standard errors of the mean (SEM).

Dose (Gy)	Maximum Erythema Score (µm)	Time after First Fraction (d)	Maximum Desquamation Score (µm)	Time after First Fraction (d)
0 (sham)	0.4 ± 0.1	12	0.0 ± 0.0	-
4 × 5	0.6 ± 0.4	18	0.6 ± 0.4	22
4 × 10	1.0 ± 0.2	20–22	2.5 ± 0.2	21
4 × 20	1.4 ± 0.3	16	2.1 ± 0.3	18
4 × 30	1.4 ± 0.1	15–16	2.7 ± 0.3	21

**Table 2:** Maximum thickness of murine ears after receiving a four-fraction course with different X-ray doses. The time of maximal ear thickness is given for every dose group. The errors are given as the standard errors of the mean (SEM).

Dose (Gy)	Maximum of Ear Thickness (µm)	Time after First Fraction (d)
0 (sham)	276.7 ± 15.0	15
4 × 5	317.9 ± 8.2	14
4 × 10	461.9 ± 17.8	22
4 × 20	524.5 ± 16.5	18
4 × 30	582.4 ± 16.9	18

**Table 3:** Semi-quantitative scores for skin erythema (top) and desquamation (bottom).

Kind of Score	Description
Erythema Score	Erythema
3	Severe
1.5	Definite
0.5	Mild
0	No
Desquamation Score	Desquamation
3	Moist
2	Crust
1	Dry
0	No
